# Dem eigenen Leben ein Ende setzen: Suizide zwischen Medizin und Ethik

**DOI:** 10.1007/s00103-021-03459-x

**Published:** 2022-01-05

**Authors:** Joseph Kuhn, Martin Härter, Peter Brieger

**Affiliations:** 1 Bayerisches Landesamt für Gesundheit und Lebensmittelsicherheit, Veterinärstr. 2, 85764 Oberschleißheim, Deutschland; 2 Zentrum für Psychosoziale Medizin, Institut und Poliklinik für Medizinische Psychologie und Institut für Psychotherapie (IfP), Universitätsklinikum Hamburg-Eppendorf, Martinistr. 52 (W26), 20246 Hamburg, Deutschland; 3 Kbo-Isar-Amper-Klinikum, Akademisches Lehrkrankenhaus der LMU, Vockerstr. 72, 85540 Haar, Deutschland

„Es gibt nur ein wirklich ernstes philosophisches Problem: den Selbstmord.“ Diese bekannte Sentenz von Albert Camus aus *Der Mythos des Sisyphos* verweist gleich mehrfach darauf, dass Suizide nicht allein unter medizinischen Gesichtspunkten zu betrachten sind. Vielmehr spielen kulturelle, ethische und rechtliche Aspekte ebenfalls eine wichtige Rolle. Bereits der Begriff des „Selbstmords“ ist ethisch brisant, weil er die aus der religiösen Tradition stammende Verneinung des Rechts auf Selbsttötung beinhaltet. Der Gegenbegriff dazu ist der „Freitod“, der die freie Verfügbarkeit über das eigene Leben hervorhebt – eine Sichtweise, die insbesondere in der aktuellen Diskussion um die Sterbehilfe hohe Aktualität hat. Doch die meisten Suizide beruhen nicht auf einer freien Entscheidung, sondern resultieren aus einer psychischen Störung oder einer existenziellen Lebenskrise. Angesichts solcher Probleme haben sich in Fachkreisen inzwischen die neutralen Begriffe „Selbsttötung“ und „Suizid“ durchgesetzt, während im allgemeinen Sprachgebrauch kaum mehr von „Freitod“, sehr wohl allerdings auch weiterhin von „Selbstmord“ gesprochen wird.

Hinsichtlich der Selbstbestimmungsfrage vertritt das Bundesverfassungsgericht den Grundsatz des Respekts vor der Autonomie des Einzelnen in der Entscheidung über das eigene Leben. Das Urteil des BVerfG vom 26.02.2020 (2 BvR 2347/15) führt dazu aus: „Das Recht auf selbstbestimmtes Sterben schließt die Freiheit ein, sich das Leben zu nehmen. Die Entscheidung des Einzelnen, seinem Leben entsprechend seinem Verständnis von Lebensqualität und Sinnhaftigkeit der eigenen Existenz ein Ende zu setzen, ist im Ausgangspunkt als Akt autonomer Selbstbestimmung von Staat und Gesellschaft zu respektieren.“

Dieser höchstrichterlichen Entscheidung wird man vielleicht in dieser allgemeinen Formulierung zustimmen können, wenn man nicht aus einer religiösen Perspektive davon ausgeht, dass der Mensch grundsätzlich nicht das Recht hat, über sein Leben zu verfügen, da dieses ein Geschenk Gottes ist. Aber auch wenn man der säkularen Sichtweise des Bundesverfassungsgerichts folgt, stellt sich die Frage, wie es sich mit diesem „Akt autonomer Selbstbestimmung“ bei Menschen mit psychischen Störungen verhält oder bei Menschen, die sich wegen einer schweren körperlichen Erkrankung das Leben nehmen wollen und nicht gut über die ihnen zur Verfügung stehenden Hilfen informiert sind, oder wenn das soziale Umfeld ihnen das Gefühl gibt, anderen nur noch zur Last zu fallen. Mit rechtlich-ethischen Erwägungen dazu schließt das vorliegende Heft. Sie sind naturgemäß nicht endgültig zu beantworten. Evident und unstrittig dagegen ist, dass der „Akt autonomer Selbstbestimmung“ kein spontaner sein kann oder sollte, sondern am Ende eines qualifizierten Beratungsprozesses steht.

Offene Fragen gibt es jedoch auch hinsichtlich der empirischen Faktenlage. 9206 Suizide verzeichnet die Todesursachenstatistik im Jahr 2020 in Deutschland. Von einer nicht unerheblichen Dunkelziffer ist auszugehen, z. B. unerkannten Suiziden unter den Verkehrstoten oder unter den Drogentoten. Ziemlich genau drei Viertel der dokumentierten Suizide entfallen auf die besondere Risikogruppe älterer Männer. Der Anstieg der Suizidrate im Alter bei den Männern wird häufig als „ungarisches Muster“ bezeichnet, weil dieser Altersgang zuerst in Ungarn beobachtet wurde. Für viele westliche Industrieländer trifft das zu, in Japan, einem Land mit insgesamt hoher Suizidrate, dagegen nicht. Dies deutet darauf hin, dass das Suizidgeschehen möglicherweise durch gesellschaftliche Altersbilder mitbestimmt wird, dass z. B. Verluste an wahrgenommener Leistungsfähigkeit und das Gefühl, nicht mehr gebraucht zu werden, das Suizidrisiko erhöhen können. Zu den vollendeten Suiziden hinzu kommt eine große Zahl an Suizidversuchen: 10- bis 20-mal häufiger schätzt die Deutsche Gesellschaft für Suizidprävention ihre Zahl. Anders als bei den vollendeten Suiziden sind hier die Frauen in der Mehrzahl und es sind mehr jüngere Menschen betroffen. Noch weitaus häufiger unter jungen Menschen sind klinisch ernst zu nehmende suizidale Gedanken, von denen nach Studien im Laufe eines Jahres bis zu einem Fünftel der Jugendlichen berichtet. Von Suiziden ist immer auch das soziale Umfeld mitbetroffen. Es leiden Angehörige, Freundinnen und Freunde, Arbeitskolleginnen und Arbeitskollegen. Man geht davon aus, dass jeder Suizid mehr als 6 Menschen mitbetrifft, von denen einige psychosoziale Unterstützung benötigen.

In der Coronakrise ist immer wieder die Frage gestellt worden, ob während des Lockdowns im Frühjahr 2020 die Häufigkeit von Suiziden und suizidalen Handlungen zugenommen hat. Dies würde der klassischen soziologischen Perspektive nach Durkheims *Le Suicide* 1897 entsprechen: In Zeiten der Anomie, der Unsicherheit über soziale Normen, nehmen Suizide zu. Das war in der Pandemieperiode 2020, wie zunächst polizeiliche Statistiken gezeigt haben, jedoch nicht der Fall. Inzwischen bestätigen auch Daten der Todesursachenstatistik diesen Sachverhalt. Aber Krisen beeinflussen durchaus die Suizidhäufigkeit, wie viele Studien zeigen: Nach der Finanzkrise 2007/2008 war das in vielen Ländern zu beobachten und auch die Coronakrise ist unter dem Gesichtspunkt der Suizidhäufigkeit mit dem Jahr 2020 nicht zu Ende, auch nicht mit dem Jahr 2021, für das noch keine Daten vorliegen. In manchen Ländern zeigt sich bereits jetzt eine Zunahme der Suizide. So haben diese in Japan seit Beginn der Krise stetig zugenommen, einer Medienmeldung im Sommer 2021 zufolge in 12 Monaten bei den Männern um 7,4 %, bei den Frauen um 18,4 %.

In der Suizidprävention gab es in den letzten Jahren in Deutschland viele Fortschritte, angefangen von der besseren Früherkennung und Therapie psychischer Störungen über Verbesserungen des Rettungswesens und der Notfallmedizin bis hin zur Entschärfung von Hotspots, etwa ungesicherten Brücken. Neue Ansätze gibt es auch in der Behandlung suizidaler Patientinnen und Patienten. Dies alles hat dazu beigetragen, dass sich die Suizidraten in den letzten Jahrzehnten nahezu halbiert haben. Dennoch bleibt hier noch viel zu tun: Lagen Mitte der 1970er-Jahre sowohl die Zahl der Verkehrstoten als auch die der Suizidopfer bei jeweils knapp 20.000 pro Jahr, so zeigt sich heute, dass die Verkehrsunfallprävention (2719 Tote im Jahr 2020) sehr viel effektiver war als die Suizidprävention (9206 Suizide 2020). So unübersehbar die Verbesserung des psychosozialen Angebots in den letzten Jahrzehnten ist, wichtige Reformvorhaben der niedrigschwelligen psychosozialen Versorgung stecken nach wie vor in den Kinderschuhen, etwa flächendeckende Angebote der Krisenintervention oder im Vorfeld gute und gut vernetzte gemeindepsychiatrische Strukturen.

Bei Camus, den wir eingangs zitiert haben, ist der Suizid eine typische Antwort auf die Sinnlosigkeit des Lebens. Er stellt dem jedoch die absurde Bejahung des Sinnlosen entgegen, in dem ebenfalls berühmten letzten Satz seines Essays: „Wir müssen uns Sisyphos als einen glücklichen Menschen vorstellen.“ Die moderne Suizidprävention geht etwas profaner davon aus, dass die meisten gefährdeten Menschen nur rechtzeitig Hilfe brauchen. Damit stellt sich die Frage, an welchen Stellen Prävention und Therapie weiter verbessert werden können, ob die Datenlage dazu ausreicht und ob für bestimmte Lebenssituationen neue ethische und rechtliche Bewertungsmaßstäbe in Rechnung zu stellen sind: Zeit also für eine Bestandsaufnahme für ein ernstes philosophisches, psychologisches, soziales und medizinisches Problem. Wir hoffen, mit dem vorliegenden Heft dazu einen Beitrag zu leisten. Den Autorinnen und Autoren danken wir dafür, dass sie uns dazu informative Artikel zur Verfügung gestellt haben, die wichtige Aspekte dieses breiten und komplexen Themenfelds vor dem Hintergrund aktueller Daten beleuchten.

